# AGHE FACULTY AWARD PRESENTATIONS AND LECTURES

**DOI:** 10.1093/geroni/igae098.0303

**Published:** 2024-12-31

**Authors:** Joann Montepare

**Affiliations:** Chair

## Abstract

The AGHE Faculty Award Presentations and Lectures will feature the Distinguished Faculty Award recipient lecture and the Rising Star Early-Career Faculty award lecture. The 2024 Distinguished Faculty Award recipient is Lynn M. Holley, PhD, FAGHE of the University of Nebraska at Omaha. This award recognizes persons whose teaching stands out as exemplary, innovative, of impact, or any combination thereof. The 2024 Rising Star Early-Career Faculty Award Lecture recipient is Yeonjung Jane Lee, PhD, of the University of Hawaiʻi at Mānoa. The Rising Star Early-Career Faculty Award acknowledges new faculty whose teaching and leadership stand out as influential and innovative.

